# Plasma 25-hydroxyvitamin D and risk of breast cancer in the Nurses' Health Study II

**DOI:** 10.1186/bcr2880

**Published:** 2011-05-11

**Authors:** A Heather Eliassen, Donna Spiegelman, Bruce W Hollis, Ronald L Horst, Walter C Willett, Susan E Hankinson

**Affiliations:** 1 Channing Laboratory, Department of Medicine, Brigham and Women's Hospital and Harvard Medical School, 181 Longwood Avenue, Boston, MA 02115, USA; 2 Department of Epidemiology, Harvard School of Public Health, 677 Huntington Avenue, Boston, MA 02115, USA; 3 Department of Biostatistics, Harvard School of Public Health, 677 Huntington Avenue, Boston, MA 02115, USA; 4 Department of Nutrition, Harvard School of Public Health, 677 Huntington Avenue, Boston, MA 02115, USA; 5 Department of Pediatrics and Biochemistry and Molecular Biology, Medical University of South Carolina, 171 Ashley Avenue, Charleston, SC 29425, USA; 6 Heartland Assays, Inc., 2325 North Loop Drive, Suite 6300, Ames, IA 50010, USA

## Abstract

**Introduction:**

Experimental evidence indicates vitamin D may play an important role in breast cancer etiology but epidemiologic evidence to date is inconsistent. Vitamin D comes from dietary intake and sun exposure and plasma levels of 25-hydroxyvitamin D (25(OH)D) are considered the best measure of vitamin D status.

**Methods:**

We conducted a prospective nested case-control study within the Nurses' Health Study II (NHSII). Plasma samples collected in 1996 to 1999 were assayed for 25(OH)D in 613 cases, diagnosed after blood collection and before 1 June 2007, and in 1,218 matched controls. Multivariate relative risks (RR) and 95% confidence intervals (CI) were calculated by conditional logistic regression, adjusting for several breast cancer risk factors.

**Results:**

No significant association was observed between plasma 25(OH)D levels and breast cancer risk (top vs. bottom quartile multivariate RR = 1.20, 95% CI (0.88 to 1.63), *P*-value, test for trend = 0.32). Results were similar when season-specific quartile cut points were used. Results did not change when restricted to women who were premenopausal at blood collection or premenopausal at diagnosis. Results were similar between estrogen receptor (ER)+/progesterone receptor (PR)+ and ER-/PR- tumors (*P*-value, test for heterogeneity = 0.51). The association did not vary by age at blood collection or season of blood collection, but did vary when stratified by body mass index (*P*-value, test for heterogeneity = 0.01).

**Conclusions:**

Circulating 25(OH)D levels were not significantly associated with breast cancer risk in this predominantly premenopausal population.

## Introduction

Vitamin D is an essential component of the endocrine system, responsible for calcium level maintenance, and is hypothesized to be involved in many other body systems. There are two circulating forms of vitamin D: 25-hydroxyvitamin D (25(OH)D) and 1,25-dihydroxyvitamin D_3 _(1,25(OH)_2_D). 25(OH)D is the main form of circulating vitamin D that is converted, by the 1α hydroxylase enzyme, into 1,25(OH)_2_D, which is considered the biologically active metabolite, binding to nuclear vitamin D receptors (VDR). However, although 1,25(OH)_2_D binds to VDR with 1,000 times the affinity of 25(OH)D, the latter is more abundant suggesting its interaction with VDR may not be trivial [1]. Although the conversion of 25(OH)D primarily takes place in the kidney, 1α hydroxylase also is present in breast and other tissues, suggesting that some portion of 1,25(OH)_2_D is produced and used locally [2-6]. 1,25(OH)_2_D is tightly regulated to maintain calcium homeostasis, while 25(OH)D serves as a pool for biologically active vitamin D and thus is a marker of overall vitamin D status [7,8].

Experimental evidence supports an important role of vitamin D in breast cancer etiology with 1,25(OH)_2_D reducing proliferation and promoting differentiation and apoptosis in breast cancer cell lines [9-11]. In animal models, vitamin D inhibited growth of both estrogen receptor (ER)+ and ER- tumors [12-14]. In a recent study in breast cancer cells, 1,25(OH)_2_D decreased the expression of aromatase [15]. Although aromatase in adipose tissue plays an integral role in estrogen levels in postmenopausal women, aromatase inhibitors may increase ovarian estradiol production in premenopausal women [16-19].

Vitamin D, which is hydroxylated in the liver into 25(OH)D, comes from dietary and supplement intake as well as sun exposure. Although several studies have assessed sun exposure and dietary intake separately, plasma levels of 25(OH)D are considered a better marker of vitamin D status because they incorporate all sources of exposure as well as skin color, weight, and other factors that influence an individual's vitamin D levels [20]. The association between circulating 25(OH)D and breast cancer has been assessed in seven prospective studies with conflicting results [21-27]. Most studies have included primarily or exclusively postmenopausal women, with the exception of a small Finnish study (*n *= 100 cases) in which plasma 25(OH)D levels measured during pregnancy were not significantly associated with subsequent breast cancer (RR = 1.4, 95% CI (0.6 to 3.4)) [25]. The inconsistent results overall and limited data in premenopausal women, coupled with the potential public health importance of vitamin D as a chemopreventive agent, prompted our assessment of this association in the prospective Nurses' Health Study II (NHSII) cohort.

## Materials and methods

### Study population

The NHSII was established in 1989, when 116,430 female registered nurses, aged 25 to 42 years, completed and returned a questionnaire. The cohort has been followed biennially by questionnaire to update exposures and ascertain newly diagnosed disease.

Between 1996 and 1999, 29,611 cohort members, who were cancer-free and between the ages of 32 and 54 years, provided blood and urine samples. These women were similar to the overall cohort with respect to lifestyle factors, such as body mass index, parity, age at menarche, past oral contraceptive use, and only differed slightly in the prevalence of family history of breast cancer (19% vs. 15% in the overall cohort) [28]. Of the 29,611 women who gave blood, 18,521 were premenopausal participants (that is, still having menstrual periods) who provided two blood samples and one urine sample timed within the menstrual cycle (one follicular sample collected on the third to fifth day and one luteal sample collected seven to nine days before the anticipated start of their next cycle). Participants were sent a short questionnaire and a blood and urine collection kit containing the necessary supplies to have blood samples drawn by a local laboratory or a colleague. Follicular plasma was aliquotted by the participants 8 to 24 hours after collection and stored in their home freezer until the luteal collection. The day of the luteal collection, follicular and luteal blood samples and luteal urine samples were shipped, via overnight courier with an ice-pack, to our laboratory where the luteal blood sample was processed and separated into plasma, and red blood cell and white blood cell components. Samples have been stored in liquid nitrogen freezers (<-130°C) since collection. The study was approved by the Committee on the Use of Human Subjects in Research at Harvard School of Public Health and Brigham and Women's Hospital. Informed consent was implied by receipt of completed questionnaires and blood samples.

### Cases

Breast cancer cases were identified on biennial questionnaires; the National Death Index was searched for non-responders. Cases had no previously reported cancer diagnosis and were diagnosed with breast cancer after blood collection but before 1 June 2007. Overall, 613 cases of breast cancer (*n *= 415 invasive) were reported on biennial questionnaires and confirmed by medical record review (*n *= 582) or verbal confirmation by the nurse (*n *= 31). Given the 99% confirmation rate on medical record review, these latter cases were included. Information on invasiveness and hormone receptor status was abstracted from the medical record. Mean time from blood draw to diagnosis was 57 months (range = 1 to 127). Two controls were matched to each case (*n *= 1,218) on age (± 2 years); menopausal status at diagnosis; month/year of collection (± 2 months); ethnicity (African-American, Asian, Hispanic, Caucasian, Other); luteal day ((date of next period-date of luteal blood draw) ± 1 day); and for each blood collection, time of day (± 2 hours) and fasting status (< 2 hours, 2 to 4, 5 to 7, 8 to 11, 12+).

### Laboratory assays

25(OH)D was assayed at Heartland Assays, Inc. (Ames, IA, USA) using a radioimmunoassay with radioiodinated tracers after acetonitrile extraction. Case-control sets were assayed together and samples were ordered randomly and labeled to mask case-control status. Samples were assayed in two batches: cases (and matched controls) diagnosed between 1999 and 2005 and those diagnosed from 2005 to 2007. The overall coefficients of variation (CVs) from masked replicate quality control samples included in each batch were 10.7% and 6.0%. Due to technical difficulties with the assay, three case-control sets failed and were removed, resulting in our final study population of 613 cases and 1,218 controls.

### Covariate data

We obtained breast cancer risk factor information from a questionnaire completed at the time of blood collection and from the biennial NHSII questionnaires. Age at menarche, weight at age 18, and height were queried in 1989. Age at first birth, parity, and diagnosis of benign breast disease were assessed biennially. Family history of breast cancer in the participant's mother and sisters was queried in 1989 and 1997. Weight was assessed at the time of blood collection.

### Statistical analysis

Quartile cut points were defined in two ways, based on the distribution in controls. The first set of cut points was calculated among all controls. Next we calculated season-specific cut points, using November to April blood collection dates to define "winter" and May to October blood collection dates to define "summer". We identified statistical outliers using the generalized extreme studentized deviate many-outlier detection approach [29]. Although we identified seven participants who had outlier values on the low end of the 25(OH)D distribution (≤3.6 ng/ml (8.99 nmol/L)), we affirmed that these were likely truly low levels given that most of these blood samples were collected in winter months and the participants had very low vitamin D dietary intake (for example, 50 to 100 IU/day). Therefore, we retained these values in the analysis.

We used conditional logistic regression to estimate relative risks (RRs) and 95% confidence intervals (CIs) to adjust for the matched design of this study. Multivariate models adjusted for body mass index (BMI) at age 18 and at the time of blood collection, ages at menarche and first birth, parity, family history of breast cancer, and history of benign breast disease. Given that outdoor physical activity may serve as a proxy for sun exposure, an important source of vitamin D, our main model did not include physical activity but we adjusted for it in a sensitivity analysis. In stratified analyses, we used unconditional logistic regression, adjusting for matching factors, since results from multivariate unconditional and conditional logistic regression models were essentially identical. To test whether the association differed by ER and PR status of the tumor, we used polychotomous logistic regression [30] with three endpoints (for example, ER+/PR+, ER-/PR-, and no breast cancer). We used a likelihood ratio test to compare a model with separate 25(OH)D slopes in each case group with a model with a common slope. Wald tests for interaction between stratification variables and hormones were used to compare the slope of the quartile medians between groups. Tests for trend were conducted by modeling quartile median concentrations and calculating the Wald statistic. The shape of the dose-response curves and tests for non-linearity were assessed using restricted cubic spline models [31,32]. We used previously published reproducibility data [33] to correct for random within-person and laboratory error [34]. All *P*-values were based on two-sided tests and were considered statistically significant if ≤0.05. All analyses were conducted using SAS software version 9 (SAS Institute, Cary, NC, USA).

## Results

Cases had slightly lower BMI at age 18 and at blood collection and had gained less weight since age 18 than controls (Table 1). Cases were more likely than controls to be nulliparous and have a family history of breast cancer and a history of benign breast disease.

**Table 1:** Characteristics of breast cancer cases and matched controls, NHSII; mean ± SD or %.

	Cases (*n *= 613)	Controls (*n *= 1,218)
Age at blood collection, y	45.0 ± 4.4	44.9 ± 4.4
Age at menarche, y	12.4 ± 1.3	12.4 ± 1.4
BMI at age 18, kg/m²	20.7 ± 2.9	21.1 ± 3.0
BMI at blood collection, kg/m²	25.5 ± 6.3	26.3 ± 7.1
Weight change since age 18, kg	11.6 ± 12.1	12.8 ± 13.8
Ever used oral contraceptives %	86	86
Duration of past oral contraceptives use, months*	47.4 ± 46.9	45.8 ± 45.6
Nulliparous, %	22	20
Parity, children^†^	2.2 ± 0.9	2.3 ± 0.9
Age at first birth, y^†^	26.6 ± 4.6	26.3 ± 4.6
Ever breast fed, %^†^	78	80
Premenopausal at blood collection, %	77	76
Family history of breast cancer, %	17	10
History of benign breast disease, %	23	16
Plasma 25(OH)D level, ng/mL (nmol/L)	25.4 (63.4) ± 9.5 (23.7)	25.0 (62.4) ± 9.6 (24.0)
Time from blood collection to diagnosis, months	57.4 ± 33.5	NA

*Duration of oral contraceptive use among ever users

^†^Among parous women

Abbreviations: SD, standard deviation; BMI, body mass index; 25(OH)D, 25-hydroxyvitamin D; NA, not applicable

In the simple conditional logistic regression model accounting for matching factors only, women in the highest quartile of 25(OH)D were not at significantly different risk of breast cancer compared with women in the lowest quartile (RR = 1.26, 95% CI (0.94 to 1.69), *P*-value, test for trend = 0.14) (Table 2). After adjustment for breast cancer risk factors, the RR was slightly attenuated (RR = 1.20, 95% CI (0.88 to 1.63), *P*-value, test for trend = 0.32), with adjustment for BMI at blood collection accounting for the majority of the difference between the simple and multivariate estimates. To assess the impact of physical activity, which could be both a confounder and a source of vitamin D, we added it to our multivariate model; results still were not significant (top vs. bottom quartile RR = 1.30, 95% CI (0.95 to 1.78), *P*-value, test for trend = 0.14). Analyses using season-specific cut points were similarly null (comparable RR = 1.06, 95% CI (0.78 to 1.44), *P*-value, test for trend = 0.79). Using restricted cubic spline models, no significant linear or non-linear associations were detected.

**Table 2:** Relative risks (95% CI) of breast cancer according to quartile of plasma 25(OH)D, NHSII.

		Quartile 1	Quartile 2	Quartile 3	Quartile 4	*P*-value, test for trend
	Cut points (ng/mL)§	< 18.4	18.4 to < 24.6	24.6 to < 30.6	≥ 30.6	
	Cases/controls	141/300	151/305	145/307	176/306	
	Simple	1.00 (ref)	1.06 (0.80 to 1.40)	1.02 (0.77 to 1.36)	1.26 (0.94 to 1.69)	0.14
	Multivariate*	1.00 (ref)	1.05 (0.79 to 1.39)	0.95 (0.71 to 1.29)	1.20 (0.88 to 1.63)	0.32
						
Invasive	Cases/controls	95/300	98/305	97/307	125/306	
	Multivariate*	1.00 (ref)	1.03 (0.74 to 1.44)	1.01 (0.72 to 1.42)	1.29 (0.92 to 1.81)	0.14
ER+^†^	Cases/controls	79/300	72/305	70/307	100/306	
	Multivariate*	1.00 (ref)	0.89 (0.62 to 1.29)	0.84 (0.57 to 1.23)	1.21 (0.84 to 1.75)	0.29
ER-^†^	Cases/controls	16/300	20/305	21/307	20/306	
	Multivariate*	1.00 (ref)	1.26 (0.63 to 2.54)	1.37 (0.67 to 2.79)	1.31 (0.63 to 2.74)	0.47
ER+/PR+^†^	Cases/controls	71/300	60/305	58/307	86/306	
	Multivariate*	1.00 (ref)	0.82 (0.56 to 1.22)	0.76 (0.51 to 1.15)	1.16 (0.79 to 1.71)	0.43
ER-/PR-^†^	Cases/controls	14/300	18/305	21/307	17/306	
	Multivariate*	1.00 (ref)	1.27 (0.61 to 2.65)	1.52 (0.73 to 3.19)	1.18 (0.54 to 2.60)	0.64

*Multivariate conditional logistic regression models adjusted for: age at menarche (< 12, 12, 13, ≥ 14 y), BMI at age 18 (< 21, 21 to < 23, ≥ 23 kg/m^2^), parity and age at first birth (nulliparous, one to two children and < 25 y, one to two children and 25 to 29 y, one to two children and ≥ 30 y, three or more children and < 25 y, three or more children and ≥ 25 y), BMI at blood collection (continuous), family history of breast cancer (yes/no), history of benign breast disease (yes/no).

Multivariate unconditional logistic regression models adjusted for above covariates and matching factors: age at blood collection (continuous), date of blood collection (February to April, May to July, August to October, November to January), fasting status at blood collection (> 8 h/≤8 h), time of blood collection (1 to 4 am, 5 to 6 am, 7 am to 12 pm, continuous), luteal day (< 8, ≥ 8, untimed collection), race (Caucasian/other), menopausal status at blood collection (premenopausal, postmenopausal, unknown menopausal status).

^† ^*P*-value, test for heterogeneity from polytomous logistic regression: 0.41 for ER+ vs. ER-; 0.51 for ER+/PR+ vs. ER-/PR-

§Cut points in nmol/L are: < 45.9, 45.9 to < 61.4, 61.4 to < 76.4, ≥ 76.4

Abbreviations: 25(OH)D, 25-hydroxyvitamin D; ref, referent; ER, estrogen receptor; PR, progesterone receptor

Analyses by tumor subtype did not show any significant differences (Table 2). When we restricted the analyses to invasive cases (*N *= 415), results were similar to the overall (top vs. bottom RR = 1.29, 95% CI (0.92 to 1.81), *P*-value, test for trend = 0.14). RRs also were similar across hormone receptor subtypes (ER+ vs. ER- *P*-value, test for heterogeneity = 0.41; ER+/PR+ vs. ER-/PR- *P*-value, test for heterogeneity = 0.51).

To assess whether the association between 25(OH)D levels and breast cancer risk differed by other factors, we conducted secondary analyses restricted to premenopausal women and women diagnosed more than two years after blood collection. Results of analyses restricted to women who were premenopausal at blood collection (*N *= 470 cases) were still null (top vs. bottom quartile RR = 1.23, 95% CI (0.87 to 1.73), *P*-value, test for trend = 0.29), as were results of analyses restricted to women premenopausal at diagnosis (*N *= 294 cases) (comparable RR = 1.40, 95% CI (0.91 to 2.15), *P*-value, test for trend = 0.16). Results also were unchanged when we excluded cases diagnosed in the first two years (RR = 1.04, 95% CI (0.74 to 1.47), *P*-value, test for trend = 0.91).

We also stratified by age at blood collection (< 45 vs. ≥ 45), BMI at blood collection (< 25 vs. ≥ 25 kg/m^2^), and season of blood collection (winter vs. summer) (Table 3). When we stratified by BMI, we observed no significant association among leaner women (top vs. bottom quartile RR = 0.90, 95% CI (0.60 to 1.33), *P*-value, test for trend = 0.45) and a significant positive association among overweight and obese women (comparable RR = 1.90, 95% CI (1.19 to 3.03), *P*-value, test for trend = 0.005). The interaction between plasma 25(OH)D and BMI was statistically significant (*P*-value, test for heterogeneity = 0.006) and a significant linear association was observed in restricted cubic spline models for BMI ≥ 25 (*P *= 0.007) but no significant association for BMI < 25 (*P *= 0.09). No non-linearity of 25(OH)D in either BMI strata was evident. Results were unchanged when stratified by age at blood collection (*P*-value, test for heterogeneity = 0.29).

**Table 3:** Stratified relative risks (95% CI) of breast cancer according to quartile of plasma 25(OH)D, NHSII.

		Quartile 1	Quartile 2	Quartile 3	Quartile 4	*P*-value, test for trend
Age at blood ≤45	Cases/controls	63/139	66/136	70/163	91/169	
	Multivariate*	1.00 (ref)	1.07 (0.69 to 1.67)	0.91 (0.58 to 1.41)	1.19 (0.77 to 1.84)	0.51
Age at blood > 45	Cases/controls	78/161	85/169	75/144	85/137	
	Multivariate*	1.00 (ref)	1.05 (0.71 to 1.55)	1.06 (0.70 to 1.61)	1.22 (0.80 to 1.85)	0.35
						
BMI < 25	Cases/controls	77/121	88/149	84/192	109/216	
	Multivariate*	1.00 (ref)	0.99 (0.66 to 1.48)	0.75 (0.50 to 1.13)	0.90 (0.60 to 1.33)	0.45
BMI ≥ 25	Cases/controls	64/179	63/156	61/115	67/90	
	Multivariate*	1.00 (ref)	1.09 (0.71 to 1.67)	1.45 (0.92 to 2.28)	1.90 (1.19 to 3.03)	0.005
						
Summer	Cut points	< 21.2	21.2 to < 27.1	27.1 to < 32.7	≥ 32.7	
	Cases/controls	73/142	70/142	73/143	71/144	
	Multivariate*	1.00 (ref)	0.95 (0.62 to 1.45)	0.99 (0.65 to 1.53)	0.97 (0.63 to 1.52)	0.95
Winter	Cut points	< 16.5	16.5 to < 22.7	22.7 to < 28.4	≥ 28.4	
	Cases/controls	71/161	83/159	78/165	94/162	
	Multivariate*	1.00 (ref)	1.22 (0.82 to 1.83)	0.98 (0.65 to 1.47)	1.22 (0.81 to 1.82)	0.52

*Multivariate unconditional logistic regression models adjusted for covariates and matching factors listed in Table 2.

*P*-value, test for heterogeneity: age at blood collection *P *= 0.29; BMI at blood collection *P *= 0.01; season of blood collection *P *= 0.52.

§Cut points in nmol/L are: Summer < 52.9, 52.9 to < 67.6, 67.6 to < 81.6, ≥ 81.6; Winter < 41.2, 41.2 to < 56.7, 56.7 to < 70.9, ≥ 70.9

Abbreviations: 25(OH)D, 25-hydroxyvitamin D; BMI, body mass index; ref, referent

Additional stratified analyses included family history of breast cancer and among blood samples collected from premenopausal women in the luteal phase of the menstrual cycle. Although there was a significant interaction by family history of breast cancer (*P*-value, test for heterogeneity < 0.001), the numbers are small and the effects in each strata of family history are not clear (for example, quartiles 2, 3, 4 vs. quartile 1 RR = 1.15, 1.14, 1.19, *P*-value, test for trend = 0.33 for no family history and RR = 0.54, 0.32, 1.49, *P*-value, test for trend = 0.31 for positive family history). Results among women with luteal phase samples were similar to overall results (data not shown).

The association between 25(OH)D and breast cancer did not vary by season of blood collection using overall cut points (top vs. bottom quartile summer RR = 1.17, 95% CI (0.74 to 1.88), *P*-value, test for trend = 0.39, winter RR = 1.23, 95% CI (0.83 to 1.84), *P*-value, test for trend = 0.44; *P*-value, test for heterogeneity = 0.33) or season-specific cut points (top vs. bottom quartile summer RR = 0.97, 95% CI (0.63 to 1.52), *P*-value, test for trend = 0.95, winter RR = 1.22, 95% CI (0.81 to 1.82), *P*-value, test for trend = 0.52) (Table 3).

When we corrected for random within-person and laboratory error using the reproducibility data (intraclass correlation coefficient (ICC) = 0.72), results were largely unchanged (for example, median of top vs. bottom quartile RR = 1.09, 95% CI (0.86 to 1.36) uncorrected and RR = 1.11, 95% CI (0.83 to 1.48) corrected).

## Discussion

In this analysis among predominantly premenopausal women, we did not observe an inverse association between plasma 25(OH)D levels and breast cancer risk overall. These results did not change when restricted to invasive cases, cases diagnosed more than two years after blood collection, or women who were premenopausal at diagnosis. Results were not substantially different between strata defined by age at blood collection or season of blood collection. Stratification by BMI yielded a significant interaction, with a significant positive association observed among overweight and obese women.

To date, only seven prospective studies of circulating 25(OH)D levels and breast cancer risk have been conducted [21-27]. Results have been inconsistent, though there have been few studies that have been able to examine this association among premenopausal women. In the NHS among mostly postmenopausal women, increasing 25(OH)D was suggestively inversely associated with breast cancer risk (top vs. bottom quintile RR = 0.73, 95% CI (0.49 to 1.07), *P*-value, test for trend = 0.06) [21]. Results from the Women's Health Initiative among postmenopausal women showed a non-significant inverse association between plasma 25(OH)D levels and breast cancer risk (top vs. bottom quintile RR = 0.82, *P*-value, test for trend = 0.20) [23]. However, other recent reports among postmenopausal women from the Prostate, Lung, Colorectal and Ovarian Cancer Screening Trial and the Cancer Prevention Study II (CPSII) showed no associations (top vs. bottom quintile RR = 1.04 (*P*-value, test for trend = 0.81), 1.09 (*P*-value, test for trend = 0.6), respectively) [22,24]. In the recent Mälmo Diet and Cancer Study, no association with total 25(OH)D was observed overall (top vs. bottom quartile RR = 0.96, *P*-value, test for trend = 0.78) and a non-significant positive association was observed among the 196 premenopausal cases (top vs. bottom quartile RR = 1.74, 95% CI (0.84 to 3.60), *P*-value, test for trend = 0.14) [26]. In contrast, the suggested inverse association with serum 25(OH)D in the E3N cohort was stronger among the small numbers of premenopausal women (*N *= 54) (top vs. bottom tertile OR = 0.37, 95% CI (0.12 to 1.15), *P*-trend = 0.11) [27]. In a small Finnish study of premenopausal women (*n *= 100 cases), plasma 25(OH)D levels measured during pregnancy were not significantly associated with breast cancer risk (top vs. bottom quartile RR = 1.4, 95% CI (0.6 to 3.4), *P*-value, test for trend = 0.4) [25]. Our current study, the largest to date among premenopausal women, shows no significant association between plasma 25(OH)D levels and breast cancer risk.

The significant interaction we observed by BMI was unexpected. Although a significant interaction by BMI also was observed in the NHS study of 25(OH)D and colon cancer, in that study, a stronger inverse association was observed among leaner women [35]. Five prior studies of the association between 25(OH)D levels and breast cancer risk, again among primarily postmenopausal women, reported no significant difference by BMI [21,22,24,26,27]. Upon further exploration, we noted that those with higher 25(OH)D levels in the BMI ≥ 25 group had higher levels of physical activity than those with lower 25(OH)D levels. Although one hypothesis is that this could reflect fewer anovulatory cycles in overweight and obese women with high vitamin D, physical activity was inversely associated with breast cancer risk in premenopausal women in this cohort and there was not a significant difference in this association by BMI [36]. Adjusting for confounding by physical activity in the analysis yielded slightly stronger RRs. Another hypothesis is that overweight women with higher 25(OH)D levels do not benefit from the reduced risk of breast cancer associated with higher BMI in premenopausal women. This is possible given that 25(OH)D levels may inhibit aromatase, which in turn could lead to increased ovarian estrogen production in premenopausal women [15-19]. It also is possible that this finding of a significant interaction is due to chance; this warrants further exploration in other studies.

The prior analysis in the NHS suggested that higher 25(OH)D levels may be associated with a lower risk of ER- or ER-/PR- disease, but no difference by hormone receptor status was observed in the CPSII cohort [21,24]. In this study of predominantly premenopausal women, we did not observe significant differences by hormone receptor status.

This study has several strengths, including the large number of premenopausal cases, extensive information on potential confounders, and blood samples collected before diagnosis. Although this analysis includes only one measure of plasma 25(OH)D to represent longer-term exposure, we have showed good reproducibility of this analyte over a two- to three-year period in the NHS (ICC = 0.72) [33].

## Conclusions

While higher plasma 25(OH)D levels have been hypothesized to reduce breast cancer risk, our results suggest such a benefit may not be observed among premenopausal women.

## Abbreviations

25(OH)D: 25-hydroxyvitamin D; 1,25(OH)_2_D: 1,25-dihydroxyvitamin D_3_; BMI: body mass index; CI: confidence interval; CPSII: Cancer Prevention Study II; CV: coefficient of variation; ER: estrogen receptor; ICC: intraclass correlation coefficient; NHS: Nurses' Health Study; NHSII: Nurses' Health Study II; PR: progesterone receptor; RR: relative risk

## Competing interests

The authors declare that they have no competing interests.

## Authors' contributions

AHE conceived of the study design, acquired and analyzed the data, interpreted the data and wrote the manuscript. DS provided statistical expertise, contributed to the analysis and interpretation of the data, and critically reviewed the manuscript. BWH assisted in the interpretation of the data and critically reviewed the manuscript. RLH performed the 25(OH)D assay and critically reviewed the manuscript. WCW and SEH conceived of the study design, secured funding, acquired the data, contributed to the analysis and interpretation of the data, and critically reviewed the manuscript. All authors read and approved the final manuscript.
